# Validity and Usability of a Smartphone Image-Based Dietary Assessment App Compared to 3-Day Food Diaries in Assessing Dietary Intake Among Canadian Adults: Randomized Controlled Trial

**DOI:** 10.2196/16953

**Published:** 2020-09-09

**Authors:** Yuwei Ji, Hugues Plourde, Valerie Bouzo, Robert D Kilgour, Tamara R Cohen

**Affiliations:** 1 School of Human Nutrition McGill University Montreal, QC Canada; 2 Department of Health, Kinesiology, and Applied Physiology Concordia University Montreal, QC Canada; 3 PERFORM Centre Concordia University Montreal, QC Canada; 4 Faculty of Land and Food Systems Food, Nutrition and Health University of British Columbia Vancouver, BC Canada

**Keywords:** mobile food record, validity, image-based dietary assessment, healthy adults, 3-day food diary, diet, application, nutrition, mHealth, Canada

## Abstract

**Background:**

Accurate dietary assessment is needed in studies that include analysis of nutritional intake. Image-based dietary assessment apps have gained in popularity for assessing diet, which may ease researcher and participant burden compared to traditional pen-to-paper methods. However, few studies report the validity of these apps for use in research. Keenoa is a smartphone image-based dietary assessment app that recognizes and identifies food items using artificial intelligence and permits real-time editing of food journals.

**Objective:**

This study aimed to assess the relative validity of an image-based dietary assessment app — Keenoa — against a 3-day food diary (3DFD) and to test its usability in a sample of healthy Canadian adults.

**Methods:**

We recruited 102 participants to complete two 3-day food records. For 2 weeks, on 2 non-consecutive days and 1 weekend day, in random order, participants completed a traditional pen-to-paper 3DFD and the Keenoa app. At the end of the study, participants completed the System Usability Scale. The nutrient analyses of the 3DFD and Keenoa data before (Keenoa-participant) and after they were reviewed by dietitians (Keenoa-dietitian) were analyzed using analysis of variance. Multiple tests, including the Pearson coefficient, cross-classification, kappa score, % difference, paired t test, and Bland-Altman test, were performed to analyze the validity of Keenoa (Keenoa-dietitian).

**Results:**

The study was completed by 72 subjects. Most variables were significantly different between Keenoa-participant and Keenoa-dietitian (*P*<.05) except for energy, protein, carbohydrates, fiber, vitamin B1, vitamin B12, vitamin C, vitamin D, and potassium. Significant differences in total energy, protein, carbohydrates, % fat, saturated fatty acids, iron, and potassium were found between the 3DFD and Keenoa-dietitian data (*P*<.05). The Pearson correlation coefficients between the Keenoa-dietitian and 3DFD ranged from .04 to .51. Differences between the mean intakes assessed by the 3DFD and Keenoa-dietitian were within 10% except for vitamin D (misclassification rate=33.8%). The majority of nutrients were within an acceptable range of agreement in the Bland-Altman analysis; no agreements were seen for total energy, protein, carbohydrates, fat (%), saturated fatty acids, iron, potassium, and sodium (*P*<.05). According to the System Usability Scale, 34.2% of the participants preferred using Keenoa, while 9.6% preferred the 3DFD.

**Conclusions:**

The Keenoa app provides acceptable relative validity for some nutrients compared to the 3DFD. However, the average intake of some nutrients, including energy, protein, carbohydrates, % fat, saturated fatty acids, and iron, differed from the average obtained using the 3DFD. These findings highlight the importance of verifying data entries of participants before proceeding with nutrient analysis. Overall, Keenoa showed better validity at the group level than the individual level, suggesting it can be used when focusing on the dietary intake of the general population. Further research is recommended with larger sample sizes and objective dietary assessment approaches.

## Introduction

Assessment of dietary intake, in particular habitual dietary intake, remains a major challenge for researchers [1]. Limitations of dietary assessment have been well documented and vary by the method chosen. Research typically relies on traditional pen-and-paper methods to assess dietary intake: the 3-day food diary (3DFD), 24-h recall, or food frequency questionnaire (FFQ) [2]. However, researchers face many challenges when deciding which method is best [2]. Issues of participant burden, motivation and willingness to accurately report diet, and participant literacy and memory should be considered. Moreover, time to enter and analyze diet data, and therefore the availability of resources to correctly analyze dietary recalls, should be accounted for before commencing a study.

All methods of dietary assessment have their limitations [3]. For instance, a single 24-h recall only reflects the food consumed from a single random day and may be less representative of an estimated individual’s intake. However, two or more 24-h recalls or food records are needed to estimate usual dietary intake distributions [4]. A limitation of food records is that they can lead to reactivity bias [5]. FFQs may lead to over reporting of average dietary intakes and, similar to 24-h recalls, rely on the participant’s ability to correctly recall portion sizes and frequencies [5]. Further, the FFQ can be lengthy; therefore, time and ability to remain focused can be demanding for participants [6]. For these reasons, new methods of dietary assessment are needed to benefit participants and researchers.

With the development of technology, image-based dietary records have increased in popularity. There are different technology-based methods of tracking diet, including taking pictures [7] or using digital food databases [2,7,8]. Relevant to this study, mobile photo meal apps engage the user to take a picture of their food item(s) using their mobile device before it is consumed and require the user to input the quantity and other pertinent details, as needed. Although mobile phone–based methods still rely on user input, an advantage of this method is that food entries are time-stamped, which identifies when food items were consumed [9]. The use of dietary assessment applications, such as those that use a mobile phone app, has shown an increase in participant satisfaction and preference compared with conventional methods (eg, 24-h recall, written food diary) [2]. Moreover, using mobile devices has the potential to reduce costs and diet data entry errors by researchers [7,10]. However, assessment of the validity of these apps against traditional methods of dietary assessment is needed to ensure participant satisfaction and therefore compliance to recording intakes.

Keenoa (Montreal, Quebec, Canada) is a smartphone imaged–based dietary assessment app that recognizes and identifies food items using artificial intelligence and permits editing of food journals in real time. Unique to other apps, Keenoa is accessed only by registered dietitians licensed to practice in Canada with the idea that dietitians are trained to identify food items that were missed or misidentified by the user. Therefore, the advantage of Keenoa is that dietitians can adjust the food items to generate accurate nutrient profiles of an individual’s dietary intake. Currently, the app is being used by practicing dietitians in Canada. From a researcher’s perspective, using Keenoa to assess dietary intakes would reduce systematic errors associated with data entry [5]. It is currently unknown if the app is appropriate for use in research.

This study aimed to assess the relative validity of Keenoa against the 3DFD and to test its usability in a sample of healthy Canadian adults.

## Methods

### Participants and Recruitment

From February to April 2019, we recruited 102 participants at the PERFORM Centre (Concordia University, Montreal, Quebec, Canada). Inclusion criteria included adults (>18 years of age) who owned a smartphone (Android or Apple) and would be able to download the Keenoa app without assistance. Exclusion criteria included individuals with a previously diagnosed disease affecting their dietary intake (ie, Type 1 or Type 2 diabetes, renal disease, inflammatory or immunity disorders); currently following a diet or weight-loss regime; who, as an adult, suffered from, had a history of, or was being treated for an eating disorder; or who have completed or are in the process of completing a nutrition or related degree (eg, a dietetics major). Finally, individuals who were not French or English speaking and who could not understand written English were also excluded. Compensation for completing the study included a detailed dietary assessment by a registered dietitian that was emailed to them. This study was approved by the University Human Research Ethics Committee of Concordia University, and written consent was obtained from all participants during their first visit at the PERFORM Centre.

### Procedure

Each participant met with a trained researcher (VB, TC) at the PERFORM Centre. Participants completed a brief sociodemographic questionnaire with questions related to total family income, ethnicity, highest level of education, age, and sex. Body weight and height were measured using a balance scale and a wall-mounted height rod, respectively. BMI was calculated using the measured weight and height (kg/m²).

Using a computer-generated list, participants were randomly assigned to start with either the Keenoa application or 3DFD for the first week and then switch methods of dietary assessment for the second week. Both 3DFD and Keenoa recordings needed to include 2 non-consecutive weekdays and 1 weekend day. Participants were highly encouraged to maintain their typical eating habits throughout the study.

Participants were provided a calendar to help them keep track of the specific days they should record their diet using the respective methods. A trained researcher also reviewed how to estimate portion sizes using the Dietitian’s of Canada Handy Guide to Servings Sizes [11]*.*

### 3-Day Food Diary (3DFD)

All participants were provided a hard-copy, single-page printout of the standard 3DFD to record their diet. On the 3DFD, there were prompts for participants to record and estimate the details of all meals and snacks they consumed including portion size, cooking methods, and any add-ons (eg, cream, oil, butter, jam). If they had meals in restaurants, participants were told that the restaurant name and dishes needed to be recorded in detail as well. Participants were given the option to either mail the 3DFD with a prestamped posted envelope directed to the corresponding author or scan and email their 3DFD to the study email. All 3DFD were coded for confidentially; those emailed were immediately printed, and the email was deleted to maintain confidentially.

### Keenoa App

Instructions on how to use the app were provided to the participant, and online tutorials were available from the company. In the case of this study, the researcher used the participant’s email to send an invitation to download the app on their smartphone, which, once downloaded, automatically connected the user to the study dietitian. Participants were asked to take pictures of the food items before they were consumed using their smartphone. Items could range from a single item (eg, an apple, French fries, a cup of coffee) to composite items (eg, a serving of lasagna, bowl of soup, or slice of pizza). If the app recognized the food item(s), it would display options for the users to choose from to correctly identify the food(s). Otherwise, participants could search and record the foods manually from a database that was linked to the Canadian Nutrient File (2015) [12]. The Canadian Nutrient File is a bilingual (English and French) food composition database that is managed by the Government of Canada that includes foods available only on the Canadian market.

Once the food item was identified, the app then prompted participants to estimate and enter the serving size. Using visual aids (ie, a tennis ball, picture of a measuring cup), the participant scrolled to the correct unit (ie, unit count, volume, or weight) and identified a number corresponding to the unit. If a participant did not consume the entire food item, they had the option to record the information in text in the app. If a participant forgot to take a picture prior to consuming a food item or meal, they also had the option to manually enter foods and text in the app.

Once a meal was complete, the user finalized the day, at which point the image and corresponding information were immediately uploaded to the research dietitians’ private page. Food items’ nutrient values were automatically computed using nutrient information from the Canadian Nutrient File (2015) database. Once participants completed their 3 days using Keenoa, the nutrient analyses were exported to Excel and coded to maintain confidentiality.

### Nutrient Analyses

All the 3DFD data were reviewed and recorded in the Food Processor software (ESHA Research version 11.1, Oak Brook, IL) by a trained researcher. The 3-day average nutrient intake content for each subject was computed by the software for total energy (kcal), fat (g), protein (g), carbohydrate (g), saturated fatty acids (g), cholesterol (g), dietary fiber (g), and micronutrients, including vitamin A (μg), vitamin B1 (mg), vitamin B2 (mg), vitamin B12 (μg), vitamin C (mg), vitamin D (μg), calcium (mg), iron (mg), magnesium (mg), phosphorus (mg), potassium (mg), and sodium (mg). All foods were entered into the software using the Canadian database, which uses the Canadian Nutrient File (2015) dataset [12]. Data were exported to Excel for statistical analysis.

The food records from Keenoa were also exported exactly as they were entered by the participant (Keenoa-participant). A research dietitian then reviewed each food record for missing items or misrecorded portion sizes as per the images (Keenoa-dietitian) and exported these data for analysis to Excel. The nutrition assessment and recommendations were sent to the participants by the research dietitian after the reports were corrected and reviewed by the dietitian. Similar to the Food Processor software (ESHA) and as mentioned, Keenoa also uses the Canadian Nutrient File (2015) database for diet analysis; the same nutrients were exported as for the 3DFD analysis.

### Exit Survey

After completing both methods of dietary assessment, the participants were sent a link to complete an online survey. This English survey included the System Usability Scale (SUS) questionnaire [13] with 3 additional questions related to using the 3DFD method and Keenoa app. The SUS has been used in previous research to examine the user’s perspective of a mobile app [14,15]. This questionnaire includes 10 items and uses a 5-item rating scale. Specifically, this questionnaire surveys participants on different aspects of the Keenoa app (eg, adoption and complexity).

### Statistical Analyses

The final analyses were restricted to those who completed both methods of diet recall (n=72), thereby excluding participants who withdrew (n=30). The characteristics of participants, as well as the results of the survey, are presented as percentages, means, and SD. Chi-square tests and Student’s *t* tests were performed to identify the differences in demographic and socioeconomic characteristics between participants who completed the study and those who dropped out. The mean and SD of the nutrition intake of the Keenoa-participant data, Keenoa-dietitian data, 3DFD data, and percentage of energy present in the macronutrients (protein, carbohydrates, and fat) were calculated.

Repeated analyses of variance (ANOVA) with post hoc tests were used to compare the differences in nutrient consumption among the 3 groups. Percentage differences (% difference) between Keenoa-participant versus Keenoa-dietitian and Keenoa-dietitian versus 3DFD were also calculated.

As the purpose of using the Keenoa app is to analyze participants’ or clients’ dietary intake after adjustment by registered dietitians, 6 cross-classification analyses were performed (ie, Pearson coefficient, cross-classification, kappa score, % difference, *t* test, Bland-Altman test) to assess the validity of the Keenoa app (Keenoa-dietitian). Weighted Cohen kappa and cross-classification tests were performed to evaluate the interrater agreement between the diet data from Keenoa-dietitian and 3DFD. This was analyzed by calculating the chance of misclassification between the 2 methods (eg, a participant being classified in the first quartile by 3DFD but classified in the fourth quartile by Keenoa or vice versa). Pearson correlations and Bland-Altman tests were also used to test for associations between Keenoa-dietitian and 3DFD. Validity assessments were performed as suggested by Lombard and colleagues [16], which combines the results of the 6 tests mentioned. The result of each test was classified as “good,” “acceptable,” or “poor,” and the total number of poor outcomes was calculated. All analyses were performed in SPSS version 23 (IBM Inc, Armonk, NY) with *P* values <.05 considered statistically significant.

## Results

We recruited 102 participants in this study; 6 individuals did not attend the baseline visit, while 21 participants did not complete the study protocol as directed (ie, using one of two methods on 3 non-consecutive days to record diet, did not complete full days of recording food items on Keenoa**,** or did not return their 3DFD as instructed.) Both 3DFD and Keenoa diet reports were completed per the study protocol by 75 participants. Due to outlying diet data that could not be edited by the system, 3 participants were excluded from the analysis. This study reports on women (n=47) and men (n=25) with a combined mean age of 38.5 years and a mean BMI of 27.0 kg/m^2^ (SD 4.9 kg/m^2^; Table 1). Most participants (80.6%) held a university degree, and 62.5% of the participants had a family income of more than Can $30,000 per year. The sample population was 56.9% Caucasian, 19.4% Asian, and 23.6% other ethnicities, including Arab, African American, and Latin American. Only 2 participants (2.8%) reported having a vegan or vegetarian diet. There was no significant difference in characteristics in those who completed the study and those who withdrew.

**Table 1:** Participant demographic characteristics (n=72).

Characteristics		Values
Age (years), mean (SD)		38.5 (16.7)
**Age (years), n (%)**		
	18-30	31 (43.1)
	31-50	18 (25.0)
	51-65	17 (23.6)
	≥65	6 (8.3)
BMI (kg/m^2^), mean (SD)		27.0 (4.9)
**BMI (kg/m^2^), n (%)**		
	<25	24 (33.3)
	25 to <30	31 (43.1)
	≥30	17 (23.6)
**Sex, n (%)**		
	Female	47 (65.3)
	Male	25 (34.7)
**Education, n (%)**		
	College and below	12 (16.7)
	University	58 (80.6)
	Refused to answer	2 (2.8)
**Family** **income (Can $), n (%)**		
	<30 000	14 (19.4)
	≥30 000	45 (62.5)
	Refused to answer	13 (18.1)
**Ethnicity, n (%)**		
	White	41 (56.9)
	Asian	14 (19.4)
	Other	17 (23.6)
**Vegetarian or vegan diet, n (%)**		
	Yes	2 (2.8)
	No	70 (97.2)

The differences between Keenoa-participant, Keenoa-dietitian, and 3DFD data are presented in Table 2. The percentage mean intakes of protein (*P*=.001), carbohydrates (*P*<.001), and fat (*P*<.001) were significantly different between Keenoa-participant and Keenoa-dietitian data as were grams of fat (*P*<.001), saturated fatty acids (*P*<.001), cholesterol (*P*<.001), vitamin A (*P*<.001), vitamin B2 (*P*<.001), magnesium (*P*=.009), phosphorus (*P*<.001), iron (*P*<.001), and sodium (*P*<.001). The majority of nutrients from Keenoa-participant were under-recorded compared with Keenoa-dietitian, excluding % protein, carbohydrates, % carbohydrates, and calcium. Vitamin A showed the highest percentage difference (134.2%, data not shown), and the lowest percentage difference was observed for potassium (0.7%, data not shown).

**Table 2:** Differences between nutrients from Keenoa-participant, Keenoa-dietitian, and 3-day food diary (3DFD; n=72).

Nutrients	Keenoa-participant, mean (SD)	Keenoa-dietitian, mean (SD)	3DFD, mean (SD)	*P* value
Energy (kcal)	1615.3 (1664.4)	1693.0 (593.2)^a^	2006.3 (540.5)	.000
Protein (g)	65.0 (40.7)	68.8 (24.9)^a^	85.6 (26.0)^b^	.000
% Protein	18.0 (5.4)^c^	16.5 (3.6)	17.4 (4.2)	.001
Carbohydrate (g)	225.5 (372.5)	181.8 (65.1)^a^	224.6 (71.8)	.000
% Carbohydrate	50.1 (12.6)^c^	43.5 (7.7)	45 (8.8)^b^	.000
Fat (g)	52.6 (29.5)^c^	77.7 (32.6)	84.9 (28.9)^b^	.000
% Fat	33.5 (9.0)^c^	37.7 (7.4)^a^	40.8 (7.5)^b^	.000
Saturated fatty acids (g)	17.5 (11.9)^c^	23.2 (10.6)^a^	27.7 (10.6)	.000
Cholesterol (g)	242.0 (190.8)^c^	283.8 (192.1)	328.3 (185.7)^b^	.000
Dietary fiber (g)	20.1 (16.6)	20.6 (8.6)	22.2 (7.8)	.323
Vitamin A (μg)	216.5 (163.8)^c^	260.6 (142.0)	270.0 (170.0)	.001
Vitamin B1 (mg)	1.1 (0.6)	1.2 (0.5)	1.3 (0.6)	.084
Vitamin B2 (mg)	1.4 (0.7)^c^	1.6 (0.7)	1.7 (0.5)^b^	.000
Vitamin B12 (μg)	2.8 (2.1)	2.9 (1.4)	3.8 (2.6)^b^	.030
Vitamin C (mg)	244.1 (901.3)	98.3 (62.4)	112.6 (62.7)	.125
Vitamin D (μg)	3.1 (2.8)	3.2 (2.0)	3.5 (3.0)	.579
Calcium (mg)	792.7 (1304.3)	691.8 (304.2)	889.4 (966.6)	.174
Iron (mg)	10.2 (5.0)^c^	11.6 (4.4)^a^	13.3 (4.1)^b^	.000
Magnesium (mg)	253.4 (146.0)^c^	278.9 (133.4)	319.2 (328.5)	.025
Phosphorus (mg)	902.1 (422.0)^c^	1023.6 (357.5)	1108.2 (370.0)^b^	.000
Potassium (mg)	2402.6 (2162.7)	2391.2 (910.7)	2553.1 (766.2)	.560
Sodium (mg)	1729.0 (1059.2)^c^	2333.9 (1203.1)	2969.0 (1621.4)^b^	.000

^a^Post-hoc tests between Keenoa-dietitian and 3DFD with *P*<.05.

^b^Post-hoc tests between Keenoa-participant and 3DFD with *P*<.05.

^c^Post-hoc tests between Keenoa-participant and Keenoa-dietitian data with *P*<.05.

The nutrient intake between Keenoa-dietitian and 3DFD were significantly different for mean intakes of energy (*P*<.001), protein (*P*<.001), carbohydrates (*P*=.001), % fat (*P*=.041), saturated fatty acids (*P*<.001), iron (*P*=.045), and sodium (*P*<.001), with fat and cholesterol showing a statistical trend (*P*=.069 and *P*=.052, respectively).

Results of the validity analysis of the Keenoa app (Keenoa-dietitian) are summarized in Table 3 and are based on the classification method by Lombard et al [16]. The highest numbers were observed for sodium and vitamin D (n=5), and the lowest numbers were observed for vitamin B1 and phosphorous (n=1). Pearson coefficient coefficients between Keenoa-dietitian and 3DFD ranged from .38 to .51 for macronutrients and .42 to .47 for micronutrients.

**Table 3:** Validity analysis of the Keenoa application (Keenoa-dietitian), based on criteria levels for good (G), acceptable (A), and poor (P) outcomes.

Nutrients	Individual level			Group level			Total number of poor outcomes	
	Association	Agreement		Agreement		Presence of bias		
	Pearson coefficient^a^	Cross-classification^b^	Kappa score^c^	% difference^d^	*t* test^e^	Bland-Altman^f^		
Energy (kcal)	A	P-G	P	A	P	P		4
Protein (g)	G	P-G	P	A	P	P		4
% protein	A	P-G	P	G	G	G		2
Carbohydrate (g)	A	P-G	P	A	P	P		4
% carbohydrate	A	P-G	P	G	G	G		2
Fat (g)	A	P-G	P	G	G	G		2
% fat	A	P-G	P	G	P	P		4
SFA^g^ (g)	A	P-G	P	G	P	P		4
Cholesterol (g)	A	P-G	P	G	G	G		2
Dietary fiber (g)	A	P-G	A	G	G	G		1
Vitamin A (μg)	A	P-G	A	P	G	P		3
Vitamin B1 (mg)	A	P-G	A	G	G	G		1
Vitamin B2 (mg)	A	P-G	P	G	G	G		2
Vitamin B12 (μg)	P	P-G	P	P	G	G		4
Vitamin C (mg)	P	P-G	P	P	G	G		4
Vitamin D (μg)	P	P-P	P	P	G	G		5
Calcium (mg)	P	P-G	P	G	G	G		3
Iron (mg)	A	P-G	P	G	P	P		4
Magnesium (mg)	P	P-G	A	G	G	G		2
Phosphorus (mg)	A	P-G	A	G	G	G		1
Potassium (mg)	P	P-G	A	G	G	G		2
Sodium (mg)	P	P-G	P	G	P	P		5
Total number of poor outcomes	7	22 (P), 1 (G)	16	4	7	9		65
Average	N/A^h^	N/A	N/A	N/A	N/A	N/A		2.95

^a^Good, *r*>.05; acceptable, *r*=.20-.49; poor, *r*<.20.

^b^Good, ≥50% in the same quartile and <10% in the opposite quartile; poor, <50% in the same quartile and ≥10% in the opposite quartile.

^c^Good, ≥0.61; acceptable, 0.20-0.60; poor, <0.20.

^d^Good, 0%-10.9%; acceptable, 11.0%-20.0%; poor, >20.0%.

^e^Good, *P* value <.05; poor, *P* value ≤.05.

^f^Good, *P* value <.05; poor, *P* value ≤.05.

^g^SFA: saturated fatty acids.

^h^N/A: not applicable.

Cross-classification results revealed that the chance of misclassification was <10% for all nutrients, except for vitamin D (misclassification rate=33.8%). Weighted kappa scores ranged from 0.000 to 0.585, with an average of 0.143. Bland-Altman plots were used to compare the differences between mean intake of each nutrient between Keenoa-dietitian and 3DFD. The results showed that the majority of nutrients were in an acceptable range of agreement, with the exception of energy (*P*<.001), protein (*P*<.001), carbohydrates (*P*<.001), % fat (*P*<.001), saturated fatty acids (*P*=.001), vitamin A (*P*<.001), iron (*P*=.03), and sodium (*P=*.005).

Finally, results from the SUS showed that the mean overall score was 61.6 points (SD 19.1 points). Data were divided and analyzed by positive (questions with odd numbers) and negative (questions with even numbers) statements. The positive statement responses ranged from “neutral” to “agree,” while the negative statement responses ranged from “disagree” to “neutral.” The majority of participants believed that Keenoa was easy to use (38/72, 52.8%) and reported they did not need the assistance of a technical person (54/72, 75.0%). There was no significant difference in acceptance between Keenoa and 3DFD (*P=*.28). However, 34.7% (25/72) of participants said that they would like to use Keenoa to track their diets, compared with only 9.7% (7/72) stating that they want to keep using 3DFD; 16.7% (12/72) would use both methods again, 27.8% (20/72) were not sure, and 11.1% (8/72) stated they would not use either method.

## Discussion

### Principal Findings

This study assessed the use of an image-based app for assessment of dietary intake in healthy adults. Similar to other studies [8,16-20], the results from Keenoa-dietitian produced similar mean nutrient profiles for more than half of nutrients when compared to the 3DFD method. However, this study included relative validation; therefore, it is impossible to conclude that one method is closer to “true dietary intake” than the other, as true dietary intake is not known.

In this study, we found significant differences between Keenoa-participant and Keenoa-dietitian data for 12 of the 22 nutrients analyzed. This suggests that the adjustments made by the dietitian were necessary to obtain the most accurate assessment of the participant’s diet. Specifically, this study found that participant reports of dietary fat and protein (% difference: +31.9% and +6.9%, respectively) were lower, while the report of carbohydrates was higher (% difference: –24.7%), compared to the edited version by the dietitian. These results suggest that Keenoa is appropriate for dietitians in clinical settings; however, dietitians should review the food entries prior to generating final reports.

Despite the advantages of image-based diet-tracking apps, food identification from the user remains a challenge unless they are highly motivated to capture all food item details. An example of this lies in proper estimation of percentages of milk fat found in dairy products that is impossible to estimate from an image unless a picture of the milk carton is taken and recorded. Items that are not easily identifiable, such as milk fat from fluid milk, become problematic if diets are high in dairy-containing sauces or are included in sandwiches and other mixed-pasta dishes such as ravioli [19-22].

We speculate this to be the case in our study, as suggested by the lower reports of dietary fat and protein. Previous studies have tried to overcome this issue by inviting participants to review their image-based food diaries with a trained researcher [23,24]. However, these studies had significantly smaller sample sizes (ie, n=40 [23] and n=20 [24]) compared to our study’s baseline participant pool (n=102), for which the time and resources needed to do these types of reviews were limited.

Compared with 3DFD, the energy and nutrient intakes reported via the Keenoa app (Keenoa-dietitian) were all low (average % difference, 22.5%). The highest % difference was observed for vitamin A (222%). When we excluded vitamin A from the analysis, the average adjustment decreased to 13%. These results are similar to that of other studies. In pregnant women (n=60), Savard et al [20] found an average 12.2% difference when comparing 3DFD with another web-based dietary assessment tool (24-h recall for 3 days). Similar to their study, we also compared a traditional dietary record method to a new app-based method; therefore, a relatively higher % difference is acceptable [16]. Nevertheless, in this study, the majority of the tests (4/7) scored poorly on 10 of the 22 nutrients when comparing the 3DFD and Keenoa-dietitian, including energy, protein, carbohydrates, % fat, saturated fatty acids, and iron. To our knowledge, only a few research groups have performed such an in-depth analysis as seen with the Keenoa-dietitian data, and although our findings cannot speak to reliability, the average number of poor outcomes is similar but slightly higher than those found by Savard et al [20] and Lafrenière et al [22].

It is suggested that a good Pearson coefficient should equal or surpass .5 [25]. Analysis in our study showed a weak association between 3DFD and Keenoa-dietitian since all values were <.5, except for protein. However, a relatively better association of energy and macronutrients between the two methods was observed with coefficient values closer to .5. Similarly, others have shown correlation coefficients between the 3DFD and web-based 24-h recall ranging from .03 to .76 [20]. A comparable trend was found in another study comparing a 4-day food record to two web-based 24-h recalls among 93 university-affiliated adults; the correlation coefficients varied between .06 and .76 [26]. Conversely, Lafrenière et al [22] observed a positive relative validity outcome with a mean adjusted correlation of .52 in their web-based 24-h dietary recall validation study. Notably, this research required participants to weigh their food and provide food labels or recipes, which may promote more accurate results. It also has been argued that a larger sample size could lead to a weaker correlation [19], since a good correlation (*r*=.46 to *r*=.93) was found by Wang et al [27] when studying a sample of 20 participants.

In order to assess the validity of Keenoa**, **the total number of poor outcomes based on 7 methods for each nutrient was counted. Among the 22 variables, only vitamin D and sodium had 5 poor outcomes, reflecting poor validity. Specifically, the vitamin D findings are similar to those found by others [20], which may be due to the fact that the majority of the Canadian population do not consume high volumes of vitamin D–enriched foods such as fatty fish and fortified dairy products daily [28]. In this study, vitamin D was the only nutrient with a higher rate of misclassification in the cross-classification analysis. By contrast, the higher SD of sodium intake indicates significant variability in average sodium intake, which could contribute to its considerable number of poor outcomes. This is understandable since sodium intake could vary from day to day. The average weighted kappa score was 0.143, representing slightly higher agreement and reliability between the two assessment tools at the individual level. This average is similar to that found by Landis and Koch [29] but is lower in comparison to the findings of Savard et al [20] and Lafrenière et al [22] who obtained average weighted kappa scores of 0.32 and 0.33, respectively. Overall, the outcomes at the group level (paired *t* tests and Bland-Altman tests) were better than those at the individual level (Pearson coefficient, cross-classification, kappa score, and % difference). These findings are supported by those of Savard et al [20] and Lafrenière et al [22] who used the same validation tests. Therefore, the validity at the group level was stronger than at the individual level, implying that the Keenoa app is more robust at analyzing group level nutrient intakes or the general population’s dietary intake. However, it should be kept in mind that bias could still exist, and the data should be interpreted carefully [16].

In this study, the average SUS score was 61.6 points, suggesting the Keenoa app “was generally well accepted; however, system users experienced usability issues” [14,30]. This is in agreement with 53.4% of participants stating that the Keenoa app was easy to use. Our results indicated that participants felt Keenoa was easy to understand and would be accepted by the general population; however, inconsistencies were found in the app, and not all participants were willing to continue using Keenoa. Indeed, compared to a written food diary, taking photos is less resource intensive, which eases the burden on participants. In line with other studies [19,31], more participants preferred using the Keenoa app over the traditional pen-and-paper method (ie, 3DFD).

Image-based dietary assessment apps, especially the Keenoa app in this study, can simplify the diet assessment process in multiple ways. Most importantly, it significantly decreases the burden on both researchers and participants by easing much of the data entry process and therefore reduces the possibility of errors [7]. In addition, because the app can be accessed remotely, it benefits people who eat out and do not take a hard copy of the food record with them [32]. Another major strength of this app is that it is linked to the Canadian Nutrient File (2015) database, which significantly reduces the data entry workload for researchers. However, some errors were still found in both Keenoa and 3DFD exported data when generating the report, which we treated as outlier data and therefore excluded those participants from this study. Although it is common to find mistakes with a new app that are often resolved with time, data should still be audited carefully in order to ensure a valid output [33]. Canada’s population consists of people with different cultural backgrounds; hence, it is hard to include every food in the database. Also, estimation error is always a challenge for image-based assessment [17]; underestimation or overestimation has frequently been observed in multiple studies. Williamson et al [34] reported that estimation error was significantly decreased by employing three analysts; however, time and budget costs are a concern with this solution. Computer-aided estimation of portion sizes is an alternative direction. In the study by Fang et al [35], a machine was able to assess the portion size with a minimum range of error. Thus, estimation error may possibly be eliminated by more capable machine learning in the future.

“Technology generation” has always been a concern with the coming of new technologies [36]. Studies with younger populations were observed to have better overall outcomes and higher usability scores compared with our study [8,17,19,31,33,37]. Younger adults are more adaptable to new technologies, while elderly users are considered to have lower adoption speeds [38-40]. However, there was no significant difference found when age was included as a covariate in our research. Thus, future research is needed to expand the validity of image-based dietary assessment to all age groups, especially the elderly.

### Limitations

Compared to other similar research [8,17,20,22], this study has relatively strong generalizability, since it included adults of different age groups, ethnicities, and both sexes. The diet data entry and analysis were all conducted by dietitians. However, this research has some limitations. First, our research has a relatively high dropout rate (27/102, 26.5%). One possible reason is that all participants only received a dietary report at the end of the study as compensation, which may have led to reduced willingness to cooperate. Future work may wish to assess self-efficacy or motivation to track dietary intake prior to commencing a study of a similar nature. However, the majority of participants who withdrew did not complete both methods, and the percentage of participants who failed to complete one of the two methods (ie, either 3DFD or Keenoa) was similar. This means that the dropout rate was more likely due to time or interest and was not related to the use of the Keenoa app itself. Second, the majority (58/72, 80.6%) of participants who completed the study held at least a university degree, which may have impacted their proficiency in using the mobile app [41]. In addition, one may argue that using the same 3-day dietary record for both methods would give a better estimation of validity. However, such an approach would lead to a higher workload for participants, and therefore, increase the drop-out rate and worsen compliance. Besides, nutrient intakes were reported by the participants in both methods. The use of unbiased reference measures, such as nutrition biomarkers or feeding studies, would have been the preferred method of validation against Keenoa; however, these are expensive. The possibility of systematic bias should be addressed when analyzing dietary intake based on images, and although 7 methods were used to assess the validity of Keenoa, we cannot eliminate the potential bias when interpreting the data. Finally, portion sizes of mixed items and sauces were challenging to estimate based on images, which might have contributed to the possibility of underestimating or overestimating the nutrient intake [19].

### Conclusion

This study assessed the relative validity of Keenoa, an image-based mobile app, against a 3DFD in healthy adults. Our results suggest that the Keenoa app has the potential to provide accurate dietary assessment information to dietitians in a more cost-efficient and time-efficient way. Furthermore, it was well-accepted by users compared with traditional methods. However, the prediction for energy, protein, carbohydrates, % fat, saturated fatty acids, and iron intake remains questionable and should be interpreted with caution. Compared to 3DFDs, Keenoa resulted in better validity at the group level than at the individual level; thereby, it may be more effective in analyzing the dietary intake of the general population. However, a study with greater representation of older adults is needed. While participants found Keenoa easier to use compared to 3DFD, further research is needed in understanding how the app can be improved from the user perspective.

## Appendix

Multimedia Appendix 1 CONSORT-eHEALTH checklist (V 1.6.1).

## Abbreviations

3DFD: 3-day food diary
A: average
FFQ: food frequency questionnaire
G: good
P: poor
SFA: saturated fatty acid
SUS: System Usability Scale
